# Cell Membrane Capacitance (*C_m_*) Measured by Bioimpedance Spectroscopy (BIS): A Narrative Review of Its Clinical Relevance and Biomarker Potential

**DOI:** 10.3390/s25144362

**Published:** 2025-07-12

**Authors:** Steven Brantlov, Leigh C. Ward, Søren Isidor, Christian Lodberg Hvas, Charlotte Lock Rud, Lars Jødal

**Affiliations:** 1 Department of Procurement & Clinical Engineering, Central Denmark Region, 8200 Aarhus, Denmark; 2 School of Chemistry and Molecular Biosciences, The University of Queensland, Brisbane 4072, Australia; l.ward@uq.edu.au; 3 Department of Clinical Medicine, SDCA—Steno Diabetes Center Aarhus, 8200 Aarhus, Denmark; soeisi@clin.au.dk; 4 Department of Hepatology & Gastroenterology, Aarhus University Hospital, 8200 Aarhus, Denmark; christian.hvas@auh.rm.dk (C.L.H.); charlotte.rud@rm.dk (C.L.R.); 5 Department of Clinical Medicine, Aarhus University, 8000 Aarhus, Denmark; 6 Department of Nuclear Medicine, Aalborg University Hospital, 8200 Aarhus, Denmark; lajo@rn.dk

**Keywords:** electrical impedance, bioimpedance, bioimpedance spectroscopy, membrane capacitance, biomarker, cells

## Abstract

Cell membrane capacitance (*C_m_*) is a potential biomarker that reflects the structural and functional integrity of cell membranes. It is essential for physiological processes such as signal transduction, ion transport, and cellular homeostasis. In clinical practice, *C_m_* can be determined using bioimpedance spectroscopy (BIS), a non-invasive technique for analysing the intrinsic electrical properties of biological tissues across a range of frequencies. *C_m_* may be relevant in various clinical fields, where high capacitance is associated with healthy and intact membranes, while low capacitance indicates cellular damage or disease. Despite its promise as a prognostic indicator, several knowledge gaps limit the broader clinical application of *C_m_*. These include variability in measurement techniques (e.g., electrode placement, frequency selection), the lack of standardised measurement protocols, uncertainty on how *C_m_* is related to pathology, and the relatively low amount of *C_m_* research. By addressing these gaps, *C_m_* may become a valuable tool for examining cellular health, early disease detection, and evaluating treatment efficacy in clinical practice. This review explores the fundamental principles of *C_m_* measured with the BIS technique, its mathematical basis and relationship to the biophysical Cole model, and its potential clinical applications. It identifies current gaps in our knowledge and outlines future research directions to enhance the understanding and use of *C_m_*. For example, *C_m_* has shown promise in identifying membrane degradation in sepsis, predicting malnutrition in anorexia nervosa, and as a prognostic factor in cancer.

## 1. Introduction

A capacitor is an electrical element that stores an electrical charge, typically by maintaining a charge difference across two close but electrically isolated surfaces. The capacity to store charge is known as capacitance. The cell membrane fulfils this function, and the cell membrane capacitance of a given tissue is denoted as *C_m_*.

Cell membrane capacitance (*C_m_*) is a key biophysical parameter that reflects the structural and functional integrity of cell membranes in all tissues. The ability to uphold a charge difference is vital in muscle, nerve, and epithelial tissues, which all rely on this electrical property to fulfil their primary physiological functions, e.g., signal transduction [1]. Overall, cell capacitance plays an essential role in the normal physiological functioning of all cell types.

Cell membranes are 5 to 10 nm thick phospholipid bilayers embedded with cholesterol, proteins, and glycoproteins. The dimensional range reflects variations depending on whether the lipid bilayer alone or the entire membrane structure is considered [1,2]. Their insulating nature enables ion pumps and channels to create electrochemical gradients of ions and other osmolytes, which are essential for cellular signalling and the maintenance of homeostasis. Channel proteins and transporters embedded in the membrane facilitate the movement of ions such as sodium (Na^+^, primarily extracellular), potassium (K^+^, primarily intracellular), and calcium (Ca^2+^, both intra- and extracellular), with concentrations typically ranging from 5 to 140 millimolar (mM) across the membrane [3]. These gradients are fundamental for a wide range of cellular processes. The gradients also represent a charge gradient across the membrane, a feature of electrical capacitors and *C_m_*.

For a given body (or other tissue), *C_m_* will be determined by the electrical properties of cell membranes, which in turn reflect the size, polarity, and charge of the membrane molecules, as well as membrane structure. Thus, *C_m_* may serve as a biomarker for assessing the state of cell membranes.

Moreover, *C_m_* degradation may act as a sentinel event in critical pathologies. In sepsis, mitochondrial membrane collapse impairs charge separation and may contribute to reduced *C_m_*, preceding organ failure [4]. While direct evidence is limited, processes such as lipid peroxidation and loss of membrane permeability, hallmarks of sepsis-induced mitochondrial dysfunction, suggest a broader breakdown of membranes that could be reflected in *C_m_*.

Understanding *C_m_* variation with physiological and pathological conditions can provide critical clinical information [5,6,7], indicating changes in cell membrane integrity due to disease, dehydration, or stress [7,8].

Practically, *C_m_* can be derived in vivo using bioimpedance spectroscopy (BIS), a non-invasive technique that analyses the electrical impedance of biological tissues across a range of frequencies. As highlighted by Lukaski [6], advances in BIS technology and modelling have enabled the extraction of physiologically meaningful parameters such as *C_m_*, offering new opportunities for clinical assessment and research.

Although this review focuses on BIS as an in vivo measurement technique, several in vitro techniques for assessing *C_m_* warrant mention. These techniques include the patch-clamp technique, electrical impedance spectroscopy (EIS), voltage clamp fluorometry, dynamic clamp, and two-electrode voltage clamp. These can offer valuable insights into membrane properties, but are primarily used in research settings [9,10,11,12,13].

The patch-clamp technique forms a tight seal between a glass micropipette and the cell membrane, allowing for the precise measurement of membrane charging and ion channel activity. EIS, in contrast, applies alternating currents across cells or tissues to extract capacitance and resistance values. Additional techniques, such as voltage clamp fluorometry, dynamic clamp, and two-electrode voltage clamp, incorporate electrical and optical measurements to probe *C_m_*. Still, their complexity limits their use in specialised experimental applications [11,12,13].

This narrative review is based on selected key studies identified through targeted literature searches and expert knowledge. Rather than systematically including all studies on *C_m_*, which range from electrophysiological measurements in isolated tissues or cells to global whole-body measurements in humans, the aim is to synthesise the underlying theory with biophysically relevant insights, with a particular focus on the clinical relevance of *C_m_* measurement in vivo. Additionally, we present the case that *C_m_* (measured using BIS) is a potential parameter for assessing cellular health. As this is a narrative review, it does not aim to provide exhaustive coverage of the literature. Nonetheless, the review aims to highlight conceptually and clinically meaningful contributions to the field. The review outlines the fundamental principles of BIS, describes the widely used and most applied biophysical model (the eponymously named Cole model) used to derive *C_m_*, and evaluates its potential utility as a clinical biomarker. In addition, it discusses the practical applications of *C_m_* in clinical settings, current challenges, and emerging opportunities, aiming to enhance its role in disease diagnosis and management.

Even though this review does not delve further into these in vitro techniques, Table 1 provides an overview of their key features, applications, and limitations for clinical use, enabling the reader to navigate between the different techniques as needed.

## 2. BIS Technology and Methodology

### 2.1. What Is BIS

BIS is a non-invasive technique that evaluates the electrical properties (impedance) of biological tissues by applying a weak alternating current (AC) to the body and measuring the resulting voltage. This process quantifies impedance (*Z*), comprising resistance (*R*) and capacitive reactance (*X_C_*), all measured in ohms. By analysing the relationship between these components over a range of frequencies, BIS provides detailed insights into body composition parameters, such as *C_m_*, measured in farad, typically as nF = 10^−9^ F.

BIS falls under the broader category of bioelectrical impedance analysis (BIA), which also includes single-frequency BIA (SF-BIA) and multi-frequency BIA (MF-BIA). SF-BIA typically measures at the fixed frequency of 50 kHz, while MF-BIA typically measures at 3–6 fixed frequencies within the range of 5 to 500 kHz [8,14]. Unlike SF-BIA and MF-BIA, which measure impedance at a limited number of frequencies, BIS measures impedance across a broad spectrum, commonly 50 to 256 frequencies, typically within the 1 to 1000 kHz range. This frequency range is selected to capture the electrical behaviour of biological tissues across what is called the beta-dispersion region, which refers to the ability of cell membranes to filter high and low frequencies, specifically, where current transitions from primarily extracellular (low frequencies) to intra- and extracellular pathways (higher frequencies). Measurement of the frequency response in the beta-dispersion range in vivo is constrained by practical considerations, including instrumentation limits, signal quality, and safety regulations related to injecting electrical currents into the body [8]. Measuring over a broad spectrum of frequencies allows BIS to differentiate between tissue types more effectively, potentially enabling the identification of changes in membrane integrity in diseases such as cancer or malnutrition [8]. The wider frequency range makes BIS superior to SF-BIA and MF-BIA for body composition analysis, including monitoring cell membrane integrity and managing diseases through early detection and treatment monitoring [8,14].

BIS devices are available in either lead-type or stand-on configurations. Lead-type devices use ECG-style electrodes (silver/silver chloride, Ag/AgCl) placed on the skin’s surface, typically the wrist and ankle. ECG electrodes offer stability and low-noise characteristics, making them commonly used in bioimpedance and electrophysiology applications. In contrast, stand-on devices combine stainless steel electrodes integrated into footplates and handles, which the user stands on and grips. By enabling whole-body and segmental measurements (arms, trunk, and/or legs), BIS devices provide flexibility in assessing specific body regions, which is especially relevant for monitoring diseases such as localised lymphedema [15] or segmental fluid shifts in patients with heart failure [16]. BIS devices are commonly known as phase-sensitive impedance devices. This means that they measure the voltage between the sense electrodes and the phase shift that occurs in the sinusoidal AC waveform. This allows the measured impedance to be resolved into its respective components, resistance (*R*) and capacitive reactance (*X_C_*). Analysis of BIS data (R and *X_C_*) at each measured frequency differentiates the contribution of extracellular and intracellular compartments to the measured impedance, allowing for the monitoring of tissue composition and hydration levels [8].

### 2.2. Capacitance and Current

As noted above, the two sides of an electrical capacitor are electrically isolated. The two sides of the electrical circuit component known as a capacitor are typically thin metal foils. In tissue, the two sides are the outside and the inside of the cell membrane. If an electrical voltage is applied across the two sides, positive electrical charge will accumulate on one side, while negative charge will accumulate on the other.

Once the accumulation has reached the level at which the charge difference corresponds to the applied voltage, current transfer can no longer occur. As a metaphor, the process can be compared to building a dam in a water stream. The flow (current) will accumulate water (charge) behind the dam (the capacitor). Once the height difference between the two sides of the dam is as high as the height difference (voltage) that was the source of the flow, water will no longer flow. This process is swift in an electrical capacitor (a tiny fraction of a second for typical capacitance and voltage); thus, a direct current (DC) cannot pass through a capacitor.

In contrast, for a swiftly changing AC, typically of sinusoidal waveform, the situation will be different. An AC will not stand still at a high level but will begin to reverse its voltage. This will cause the charge to flow again, this time in the opposite direction, until a corresponding charge difference in the opposite sign has been established, at which time things will halt again. In the metaphor, this corresponds to a (strange) landscape that lowers at the high side and elevates at the low side, causing the stream to flow in the opposite direction until the reservoir has been filled, but this time at the other side of the dam, followed by another shift, and so on.

AC changes direction with a given frequency. If the frequency is low (compared to the time it takes for charge to accumulate in the capacitor), the capacitor will block current flow, except for brief moments around the shifts. But if the frequency is very high, the current will run back and forth as an AC sine wave. No individual charge will pass the capacitor (the cell membrane). Still, the continual charge flow to and from the two surfaces will correspond to an alternating current as a collective phenomenon.

As an interesting phenomenon, the voltage and the current across a capacitor are not in phase. When the voltage reaches its peak value, the accumulated charge is also at its peak. This means that there is no longer an accumulation of charge, i.e., no current flows at that instant. As the voltage falls, the current flows in the opposite direction. The current reaches its maximum when the voltage crosses zero, shifting from positive to negative, and similarly for the negative half of the cycle. This phase shift corresponds to a quarter-cycle displacement between the voltage and the current waves, known as a 90° phase shift.

### 2.3. Impedance and Phase Angle

BIS devices measure the impedance (*Z*) of tissue, which is a combination of its resistance (*R*) and capacitive reactance (*X_C_*). The resistance arises from the conductive properties of the tissue fluids, while the reactance arises from the cell membrane capacitance—both as an overall effect of the combined tissue (to be described below).

Generally, an impedance *Z* which is a combination of resistance *R* and reactance *X_C_* can be represented by a complex number:

$$Z=R+jX_{C}$$

where *j* is an imaginary unit (*j*^2^ = −1) which represents a 90° phase shift between current and voltage. For the combined expression, the phase shift is generally much lower than 90°.

*Z* can be represented as a vector in a coordinate system, with *R* on the horizontal axis and *X_C_* on the vertical axis. The *phase angle* is then the angle between the vector and the horizontal axis. The magnitude of the total impedance is the length of the vector:

$$\left|Z\right|=\sqrt{R^{2}+X_{C}^{2}}$$

### 2.4. Electrical Models for Tissue

In biological tissues, current can flow through cells (intracellular pathway) or around cells (extracellular pathway), or a combination of both. The extracellular pathway involves conduction through the extracellular water (ECW). The intracellular pathway involves crossing the cell membrane (capacitance) and conduction through the intracellular water (ICW).

**Figure 1:**
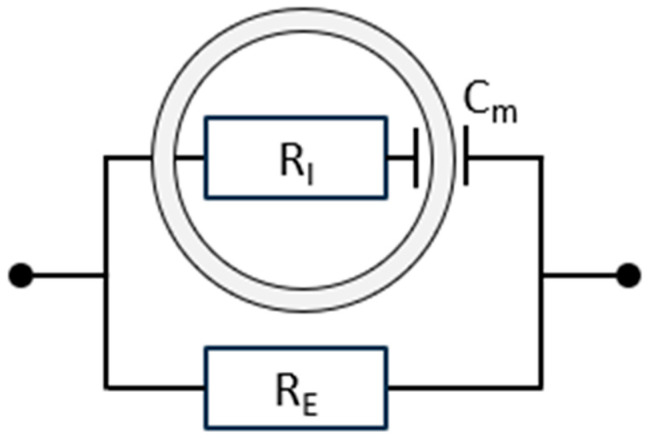
A simple equivalent parallel electrical circuit for biological tissue, representing two distinct components. The upper branch models the intracellular pathway, where current crosses the double-layered cell membrane (electrically represented by the capacitance *C_m_*) and passes through the intracellular water (ICW, electrically represented by the resistance *R_I_*). The lower branch represents the extracellular compartment, characterised by *R_E_*, the resistance of the extracellular water (ECW). More complex models have been developed, but these provide minimal practical advantage [2].

BIS models biological tissues using an electrical circuit that captures both conductive and capacitive properties (see Figure 1). Resistors (*R*) represent conductive pathways: *R_E_* denotes the resistance of the ECW, and *R_I_* denotes the resistance of the ICW, both measured in ohms (Ω). Owing to its charge storage capacity, the cell membrane is modelled as a capacitor (*C_m_*) [2,6,8]. As will be elaborated in Section 2.7, the model shown in Figure 1 represents a simplification of reality, but a very useful simplification for practical applications of BIS.

The impedance *Z_RC_* of the serial connection of *R_I_* and *C_m_* (the intracellular pathway) is represented by the complex number

$$Z_{RC}=R_{I}-j\frac{1}{\omega C_{m}}$$

In this expression, 1/*ωC_m_* is the capacitive reactance, *X_C_*, of the cell membrane capacitance, where *ω* = 2π*f* is the angular frequency of the applied AC signal. Note that the reactance decreases as the frequency increases.

The impedance of the extracellular pathway is the resistance *R_E_*.

The BIS device measures total impedance, which is a parallel combination of *Z_RC_* and *R_E_*, which can be calculated by the equation

$$\frac{1}{Z_{Total}}=\frac{1}{R_{E}}+\frac{1}{Z_{RC}}$$

which can be rewritten as follows:

$$Z_{Total}=\frac{Z_{RC}\cdot R_{E}}{Z_{RC}+R_{E}}$$

It should be noted that *Z_RC_* is a complex number (which depends on the AC frequency). Disentangling the expression into real components unfortunately leads to somewhat complicated expressions (some of which will be presented later).

The parallel model is particularly suitable for bioimpedance spectroscopy as it reflects physiological current pathways more accurately than series models. For an in-depth discussion of parallel vs. series configurations in tissue modelling, see Foster & Lukaski [17].

### 2.5. Frequency Dependence and BIS

**Figure 2:**
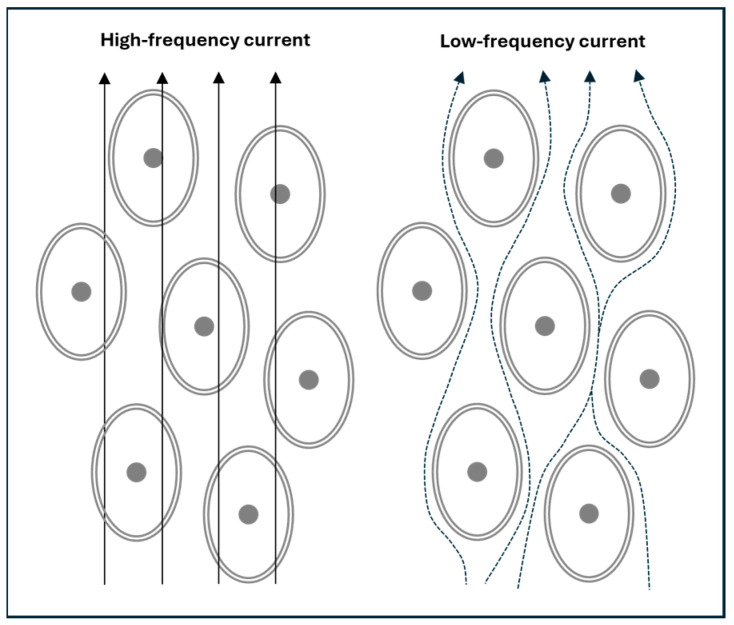
A simplified depiction of electric current flow in body tissues. At high frequencies, the current travels through both the extracellular water (ECW) and intracellular water (ICW), collectively representing the total body water (TBW). In contrast, low-frequency currents cannot cross cell membranes and are confined to the ECW.

At low frequencies (e.g., 5 kHz or lower), the cell membrane’s capacitive reactance (giving rise to *X_C_*) is high, which restricts current flow primarily to the extracellular compartment (*R_E_*). As frequency increases, *X_C_* decreases progressively, allowing more current to pass through the cell membrane and into the intracellular compartment (*R_I_*) and its associated capacitance (*C_m_*). At high frequencies (e.g., above 100 kHz), the current effectively penetrates the cell membrane, enabling flow through the ECW and ICW fluid spaces [6]. This frequency-dependent behaviour of current flow is illustrated in Figure 2.

The transition from low to high frequencies is not abrupt but follows a continuous spectrum: membrane penetration is minimal at near-zero frequencies and becomes nearly complete only as frequencies approach infinity. This frequency-dependent behaviour underlies the principle of BIS.

The interplay between *R_E_*, *R_I_*, and *C_m_* governs the frequency-dependent impedance measured by BIS, reflecting how current flows through and around cells across frequencies.

### 2.6. The Cell Membrane as a Parallel-Plate Capacitor

**Figure 3:**
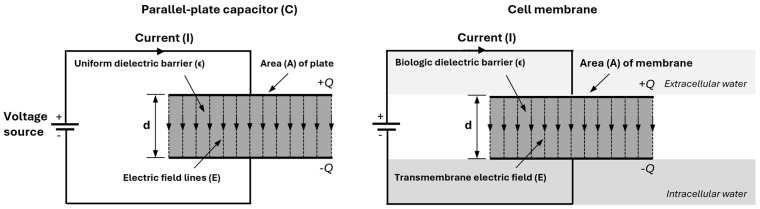
A parallel-plate capacitor and a biological cell membrane are both capacitors. The capacitor (**left**) stores charge (+*Q*, −*Q*) across a dielectric barrier, generating a displacement current. Its capacitance is *C* = *ϵA*/*d*, where *ϵ* is the dielectric constant, *A* is the plate area, and *d* is the plate separation. Similarly, the cell membrane (**right**) acts as a capacitor, with the phospholipid bilayer as the dielectric barrier.

In a parallel-plate capacitor, the two surfaces are separated by a dielectric material, namely the phospholipid bilayer of the cell membrane (see Figure 3) [18,19]. The relation between charge *Q* (in coulombs), capacitance *C* (in farads), and voltage *U* (in volts) is

Q=C·U

The capacitance of a parallel-plate capacitor is determined by plate area *A* and the distance *d* between the plates:

$$C=\frac{\epsilon A}{d}$$

While cell membrane capacitances follow the same capacitance relationship, the biological membrane is structurally more complex than a simple plate, containing embedded proteins and ion channels. When BIS measures *C_m_*, the measurement reflects properties related to cellular integrity and composition as well as total membrane area.

### 2.7. The Biophysical Cole Model Used in BIS

As described above (Section 2.4), BIS models biological tissues using an equivalent electrical circuit that captures both the conductive and capacitive aspects of the tissue. The Cole model, used by many researchers to analyse data obtained from BIS devices, provides the theoretical foundation for understanding the electrical properties of biological tissues through bioimpedance measurements as functions of frequency [8,20,21].

If the impedance is calculated based on the simple electrical model from Figure 1, it can be shown that the complex values of the impedance $Z=R+jX_{C}$ will fall in a half-circle on an (*R*, *X_C_*) plot, with the centre of the half-circle on the *R* axis. In practice, however, BIS measurements show a slightly different pattern: while the half-circle is a very good approximation to the data (at least in the frequency range from a few kHz up to 1 MHz), the centre is not on the *R* axis. A better representation of the reality of bioimpedance can be given by replacing the *R_I_*–*C_m_* branch with the more generalised concept of a constant phase element [22].

**Figure 4:**
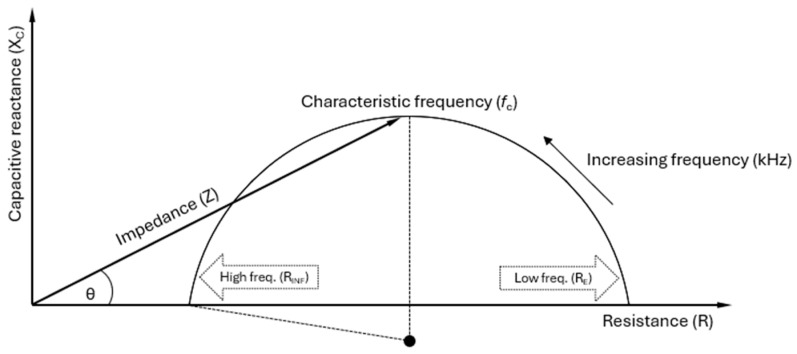
A Cole plot illustrating the impedance (*Z*) of biological tissue as a combination of resistance (*R*) and capacitive reactance (*X_C_*), varying with frequency. Strictly, the reactance *X_C_* of a capacitor is negative, but by convention, the Cole plot is plotted with *X_C_* as positive [23]. The impedance vector forms a phase angle (*θ*) with *R*, indicating the phase shift between the applied voltage and current. The rightmost point (*R_E_* or *R*_0_) represents low-frequency resistance, reflecting the extracellular water (ECW). In contrast, the leftmost point (*R_INF_* or *R*_∞_) corresponds to high-frequency resistance, encompassing both the ECW and intracellular water (ICW), indicative of the total body water (TBW). The characteristic frequency (*f*_c_) at the plot’s peak marks where cell membrane capacitance (*C_m_*) significantly affects current flow, with *R_E_* in parallel with *R_I_* and *C_m_*, as shown in Figure 1. Due to distribution effects (e.g., cell size and shape), biological tissues exhibit alpha dispersion factor (*α*) values between 0 and 1. Mathematically, *α* < 1 causes the centre point of the semi-circle to be suppressed below the *R* axis [8].

The Cole model handles this by conceptually keeping the simple electrical model from Figure 1 while introducing a dimensionless parameter *α* (0 < *α* ≤ 1) that causes the centre of the circle to deviate from the *R* axis, as shown in Figure 4.

Although not perfect, the Cole model is widely regarded as the best for analysing the impedance (*Z*) of biological tissues by quantifying both resistive (resistance) and capacitive (reactance) electrical parameters [2,8]; to quote Grimnes and Martinsen, “*The task is to choose a productive model, for example, as simple as possible but not too simple, allowing conclusions and predictions*” [24].

By analysing the measured impedance data (resistance and reactance) across a range of frequencies using the Cole model, key parameters such as *R_E_* (also written *R*_0_), *R_INF_* (also written *R*_∞_), *C_m_*, *τ*, and *α* can be extracted. These parameters describe the electrical behaviour of tissues and are directly relevant to clinical applications [8]. The Cole model links theoretical biophysics to practical health assessment by relating these parameters to clinical applications, but its use depends on the specific clinical and research context [6,15].

The formula used in the Cole model to describe the impedance (*Z*) and the relation to the parameters presented above is

$$Z\left(f\right)=R_{\infty }+\frac{R_{0}-R_{\infty }}{1+{\left(j\omega \tau \right)}^{\alpha }}$$

Below is an overview of the involved parameters:

- *Z*(*f*): The impedance, or total opposition to the flow of AC at a particular frequency (*f*), is measured in ohms (Ω). Impedance combines both resistive and reactive elements. Through *ω* (see below), *Z*(*f*) varies with frequency, i.e., the impedance is frequency-dependent.
- *ω*: The angular frequency of the applied alternating current, related to the frequency *f* by the formula *ω* = 2π*f*, which converts frequency to angular frequency in order to standardise formulas for analysing waves and oscillations. As frequency increases, the capacitive effects decrease, affecting the total impedance.
- *R*_0_ or *R_E_*: The resistance at zero frequency (*f* = 0), i.e., under direct current (DC) conditions. The cell membranes act as insulators at zero or low frequencies (in practice, typically <5 kHz), allowing current to flow primarily through the ECW. As a result, R_E_ reflects the electrical resistance of the ECW, where the capacitive effect of the cell membrane significantly limits current flow into the intracellular space, leading to higher resistance.
- *R*_∞_ or *R_INF_*: The resistance at infinite frequency (*f* = ∞). At very high frequencies, the capacitive reactance of the cell membrane becomes negligible, allowing current to pass through both the ECW and the ICW compartments. This is due to the rapid oscillations that make the cell membranes permeable to the AC. Therefore, *R_INF_* reflects the total resistance of the TBW, the combination of the ECW and ICW. Since the current has more pathways to travel, *R_INF_* is lower than *R_E_*, where the current is restricted to the extracellular space.
- *R_I_*: The resistance of the ICW, which is related to *R*_0_ = *R_E_* and *R*_∞_ = *R_INF_* by the formula
  $$R_{I}=\frac{R_{0}\cdot R_{\infty }}{R_{0}-R_{\infty }}$$
- *j*: The imaginary unit in the complex numbers, defined by the property that *j*^2^ = −1. It is used in the equation to represent the phase shift introduced by the capacitive element in the system. In the impedance context, *j* distinguishes between the resistive (real) and reactive (imaginary) components. If the complex impedance *Z* = *R* + *jX* is visualised as a vector in the complex plane, with *R* on the horizontal axis and *X* on the vertical axis, the angle between the vector and the horizontal axis represents the phase difference between voltage and current.
- *τ* (tau): The time constant, representing how quickly tissue responds to an AC signal. It shows how different tissue components, such as cell membranes, accumulate and release electrical energy. The time constant is *τ* = (*R_E_* + *R_I_*) ⋅ *C_m_*, where *C_m_* is the cell membrane capacitance. *τ* indicates the characteristic time over which the system adjusts to changes in the electrical field. Bioimpedance helps determine the speed at which the tissue reacts to the applied alternating current, providing insights into the electrical behaviour of the tissue.
- *f*_c_: The characteristic frequency is the point at which the capacitive properties of the cell membrane are most pronounced, meaning the reactance (the imaginary part of the impedance), reaches the peak (or top point) of the semi-circle in the Cole plot. The membrane’s capacitance impedes current flow at this frequency, making it crucial in clinical measurements. The characteristic frequency maximises the membrane’s ability to accumulate and release electrical charge, providing critical insights into membrane functionality in bioimpedance analysis. *f*_c_ can be mathematically calculated as
  $$f_{c}=\frac{1}{2\pi \cdot C_{m}\cdot \left(R_{E}+R_{I}\right)}$$
- Clinically, *f*_c_ is essential for understanding how the cell membrane behaves under alternating current. It represents the transition point between low-frequency resistance dominance and high-frequency capacitive dominance, offering insight into the cell membrane’s electrical properties.
- *α*: A parameter allowing the adjustment of the Cole plot. The value *α* = 1 corresponds to leaving out *α* from the formula for *Z*(*f*), which places the centre of the semi-circle on the *R* axis. This is the expected behaviour of the idealised circuit shown in Figure 1. In non-ideal reality, the Cole plot has a centre slightly below the *R* axis (see Figure 4), reflecting that the body consists of more than one type of tissue and therefore a mixture of relaxation times [8]. This is mathematically modelled with values of *α* < 1, with *α* ≈ 0.7 as a typical value.

The formula for *f*_c_ can be rearranged to isolate and calculate *C_m_*. At *f*_c_, the capacitance of the cell membrane maximally impacts the impedance, corresponding to the top point of the semi-circle in the Cole plot. This critical point is essential for accurately assessing *C_m_*. The formula used is

$$C_{m}=\frac{1}{2\pi {\cdot f}_{c}\cdot \left(R_{E}+R_{I}\right)}$$

## 3. Physiology and *C_m_*

### 3.1. Factors Influencing C_m_ and Its Interpretation

Several factors affect *C_m_* in biological membranes. Membrane thickness, for instance, influences capacitance, as thicker membranes (usually 7.5 to 10 nm) reduce *C_m_* by increasing charge separation.

The composition of cell membranes, particularly cholesterol content and phospholipid types, may influence charge storage by affecting membrane stiffness [25]. The precise impact of cholesterol on *C_m_* remains unclear, requiring further research to clarify this relationship, which is beyond the scope of this review. Membrane integrity also plays a role: healthy, intact membranes tend to have predictable *C_m_* values, typically in the range 0.5–1.0 µF/cm^2^, whereas damaged membranes often exhibit altered capacitance due to structural compromise [18]. The quoted values for µF/cm^2^ refer to the capacitance per membrane area of the *individual* cell. They cannot, in a simple way, be used to calculate the *collective* value of *C_m_* (capacitances are not just added). Still, the overall *C_m_* will reflect what is going on at the micro-level.

Changes in *C_m_* measured by BIS can therefore serve as biomarkers of membrane health. Increased capacitance may indicate reduced membrane thickness, while lower *C_m_* may suggest membrane thickening or structural damage. Furthermore, BIS measures impedance across various frequencies, allowing for the modelling of extracellular and intracellular compartments.

Although *C_m_* derives from the electrical properties of the cell membrane, *C_m_* may also be influenced by the total membrane area exposed to the measuring current. For example, muscle hypertrophy resulting from physical exercise will, to some extent, cause an expansion of the muscle cell area, which could influence *C_m_* in ways unrelated to membrane structure. The question of relations influencing *C_m_* warrants further investigation.

In typical BIS applications, *C_m_* is calculated for the whole body or a segment containing a heterogeneous mix of cell and tissue types. The measured *C_m_* likely represents a weighted average of the membrane capacitance across all cell types along the conductive path. This averaging makes interpreting *C_m_* in a tissue- or cell-specific context difficult. The following section explores how *C_m_* may yield clinically meaningful insights despite these limitations.

These physiological variations, linked to body size, tissue composition, and organ-specific cellular density, can contribute to inter-individual variability in measured *C_m_*. For example, individuals with greater lean body mass may exhibit higher *C_m_* values simply due to a larger aggregate membrane surface area, independent of cellular dysfunction. Likewise, hydration status may influence conductivity and membrane polarisation, subtly altering the derived *C_m_*.

It should be noted, however, that *C_m_* is also likely to be influenced by body geometry and fluid distribution, as is the case with other measures, such as phase angle (*PhA*) and resistance ratio (*R_E_*/*R_I_*). Therefore, the careful interpretation of *C_m_* requires the consideration of underlying anthropometric and compositional factors, especially when comparing across populations or disease states.

### 3.2. C_m_ in Disease Monitoring

Measuring *C_m_* with BIS in clinical practice reflects the capacitance of muscle, fat, nerve, epithelial, endothelial, and cancer cells, giving an overall view of the membrane health in the analysed tissue. Although the clinical use of *C_m_* is still limited, early findings suggest its potential across a range of conditions. Its ability to reflect membrane integrity and functional cell status makes it a relevant candidate for both diagnosis and monitoring. Expanding the clinical framework for *C_m_* could help translate these early observations into meaningful applications.

Healthy cell membranes exhibit higher capacitance, and damaged membranes show reduced capacitance, indicating potential dysfunction [2,6,23,26].

High *C_m_* has been linked to insulin resistance in healthy adults, but not to metabolic syndrome without insulin resistance, suggesting that *C_m_* could be a specific, non-invasive indicator of insulin resistance [27]. In healthy individuals, *C_m_* and *f*_c_ are critical to surface electromyography (EMG) and muscle contractile properties, likely due to T-tubules [28]. In patients with lymphedema, differences in *C_m_* align with known variations in lymphedema types [29]. Overall, increases in *C_m_* may indicate changes in body composition, tissue homogeneity, and total cell mass, suggesting its potential as a marker for nutritional status and prognosis in patients on haemodialysis [30].

Exploring *C_m_* in the diagnosis and treatment monitoring of cancer offers the potential for proxy information on tumour progression and therapeutic response. Notably, *C_m_* has been identified as a significant, independent predictor of survival in patients with advanced head and neck cancer beyond established prognostic factors [26]. Similarly, investigating its association with cardiovascular health could facilitate the early detection and management of heart disease [31,32]. In patients on dialysis, *C_m_* measurements may enhance the optimisation of treatment protocols by providing insights into kidney function and fluid balance [30].

In children with nephrotic syndrome, changes in *C_m_* have been shown to reflect disease status, indicating affected cell membrane properties [33]. In younger patients (<18 years) with anorexia, *C_m_* was significantly higher than in older patients, possibly reflecting age-related differences in membrane properties. *C_m_* also increased with treatment, indicating improved nutritional status [34].

In patients with gastrointestinal disease, *C_m_* was lower in those with more significant weight loss, suggesting its potential as a marker of malnutrition [35].

Differences in *C_m_* trends across conditions likely reflect distinct underlying mechanisms. In anorexia [34], elevated *C_m_* may result from changes in membrane lipids or ion channel density during starvation and refeeding. In contrast, reduced *C_m_* in cancer may reflect membrane disruption, apoptosis, or the loss of viable cell mass, where lower *C_m_* is associated with worse clinical prognosis [26].

Based on the current knowledge of *C_m_*, this suggests that *C_m_* alterations vary by disease, tissue status, and membrane remodelling.

These observations, together with findings in other clinical conditions such as cancer, anorexia nervosa, and nephrotic syndrome, are summarised in Table 2.

In clinical BIS, the *C_m_* derived from whole-body measurements represents an apparent or notional capacitance, not a direct biophysical measurement of true membrane capacitance at the cellular or subcellular level. This distinction is essential when interpreting clinical data: the “tissue” under study in this context is the entire body, and *C_m_* reflects aggregated membrane behaviour that may have prognostic relevance [8].

### 3.3. C_m_ and Other BIS Parameters

Other raw impedance parameters are used to study cellular properties. Among these, capacitive reactance (*X_C_*) and phase angle (*PhA*) at 50 kHz are widely applied, as they provide complementary information about tissue electrical properties [23,36]. While both *X_C_* and *PhA* provide insights into tissue electrical properties, *PhA* is particularly associated with cell mass, hydration, and membrane integrity, and has demonstrated clinical relevance in diagnostics and research [23,36,37].

Table 3 outlines *C_m_*, *PhA*, and *X_C_* and their definitions, roles, and differences, which are essential for their correct use and interpretation. While *PhA* and *X_C_* can be measured with all types of BIA techniques, *C_m_* measurement requires a BIS technique [8]. Together, these parameters contribute to understanding cellular behaviour and integrity. *PhA* at 50 kHz and *C_m_* are closely related. *C_m_* is defined as the capacitive reactance at the characteristic frequency. On average, *f*_c_ is close (43.8 kHz) to 50 kHz, but the variation is large [23]. When evaluating cell membrane properties, *f*_c_ and *C_m_* appear more directly linked than *PhA*. However, further research is needed to assess the benefit of using *C_m_* rather than *PhA* at 50 kHz.

Compared to *PhA* and *X_C_*, *C_m_* may provide a more specific and mechanistic assessment of membrane structure and function. While *PhA* reflects a combined signal of resistance and reactance at a fixed frequency, *C_m_* is derived from the full frequency spectrum and mathematically isolates the capacitive contribution of the membrane. This may allow *C_m_* to detect earlier or more subtle changes in membrane status that do not yet affect cell mass or hydration. Thus, *C_m_* offers complementary and potentially more-targeted information in specific clinical or research settings.

Theoretically, it is possible to estimate *C_m_* using MF-BIA devices, which typically measure 3–6 frequencies within the 5 to 500 kHz range. However, although MF-BIA can apply the Cole model for an estimate, it offers a more limited frequency spectrum than BIS, raising concerns about its extra- and intracellular resistance (*R_E_* and *R_I_*) estimates.

Currently, no definitive conclusions can be made about the accuracy of *C_m_* estimations using MF-BIA without further studies that directly compare *C_m_* values obtained from MF-BIA and BIS. Such research is necessary to establish the validity of MF-BIA as an alternative approach for *C_m_* measurement and to determine the extent to which it correlates with the BIS technique. Until such studies are conducted, the use of MF-BIA for accurate *C_m_* estimation remains theoretical.

### 3.4. Limitations and Future Perspectives

As noted in Section 2.7, the concept of a constant phase element (CPE) appears to be a more correct description of the bioelectrical reality than a single *C_m_* value in series with resistive values (*R_I_*). In practice, however, the Cole model handles this well by introducing the parameter α. This is an area full of interesting theoretical issues, but we are not aware of clinical applications that go beyond the Cole model, although alternative models have been used in pre-clinical animal studies [22].

Although *C_m_* has potential as a biomarker for cellular health, several limitations hinder its broader clinical application. Research remains limited due to the challenges associated with its measurement and interpretation, as well as the more restricted availability of BIS equipment in clinical and research settings [38].

Compared to SF-BIA, BIS devices are considerably more expensive, limiting their availability to well-funded research institutions and specialised clinical centres. The lack of standardised measurement protocols makes clinical application challenging, highlighting the need for clear guidelines to ensure reliable and comparable measurements.

It is unclear to what extent existing *C_m_* studies have been standardised, making comparisons and clinical interpretation difficult. Future research should assess standardisation levels and establish protocols covering electrode placement, patient positioning, environmental conditions, device calibration, and validated interpretation techniques. Additional critical factors must also be identified to enhance measurement accuracy and consistency [39].

Research across diverse populations is needed to clarify the clinical relevance of using *C_m_* and reinforce its role in diagnostics and prognostics. Large-scale studies are necessary to establish normative data and reference ranges [40]. Addressing these gaps is needed to fully realise the potential of *C_m_* in clinical and research settings.

Future research should explore new clinical applications of *C_m_* across various medical fields, focusing on how to integrate *C_m_* measurements with other diagnostic tools and biomarkers to enhance diagnostic accuracy and patient management. A multimodal approach combining bioimpedance data with imaging, molecular, and biochemical markers can offer comprehensive health assessment for patients with complex morbidities, such as those with cancer and chronic inflammation.

Emerging impedance-related approaches include AI-based *C_m_* pattern analysis, wearable BIS devices, and multi-omics platforms. The field is still new, and the right balance between patient and consumer protection and regulatory hurdles has likely not yet been found; medical devices and software are regulated by the FDA in the United States, and CE marking in Europe includes requirements under the EU Medical Device Regulation (MDR) [41,42].

## 4. Conclusions

Cell membrane capacitance, *C_m_*, represents a promising parameter for assessing cellular health and integrity, with potential clinical relevance across various fields. Measured non-invasively through BIS, *C_m_* may provide insights into membrane structure and function, with higher values typically associated with intact, functional membranes and lower values reflecting possible dysfunction.

While early findings are encouraging, the clinical application of *C_m_* remains at an exploratory stage, and the current evidence does not yet permit definitive conclusions about its diagnostic or prognostic value. Further validation and broader adoption in clinical settings face challenges, including limited clinician awareness, variability in measurement protocols, and the need for population-based reference data.

Future research should involve close collaboration between bioimpedance scientists and clinicians, focusing on elucidating disease-specific patterns, pathophysiological mechanisms, and the added value of *C_m_* relative to established bioimpedance parameters.

Emerging tools, such as AI-based *C_m_* analytics, wearable BIS devices, and multi-omics integration, offer exciting opportunities for advancing *C_m_*-based diagnostics, but both technical and regulatory issues remain.

Systematic studies and standardised protocols will be essential for evaluating the reliability and utility of *C_m_* as a biomarker. With these efforts, *C_m_* may evolve into a clinically meaningful complement to existing bioimpedance metrics.

## Figures and Tables

**Table 1:** An overview of techniques for measuring membrane capacitance (*C_m_*), highlighting their invasiveness, resolution, clinical use, and limitations. Some techniques are used in routine clinical practice. Others are restricted to basic electrophysiological research (e.g., ion channel studies in isolated cells) or experimental settings (e.g., early-stage testing not yet established in clinical care), such as cancer detection, wound monitoring, neurological research, and tissue engineering.

Technique	Invasiveness	Resolution	Clinical Use	Limitations
Bioimpedance spectroscopy (BIS)	Non-invasive	Whole body/ segmental	Routine use	Requires calibration, affected by hydration, temperature, and body position
Patch-clamp	Invasive	Single-cell level	Research (electrophysiology)	Requires isolated cells and skilled operators, unsuitable for in vivo use
Electrical impedance spectroscopy (EIS)	Semi-invasive	Tissue-specific	Research/ experimental	Limited clinical use; sensitive to electrode setup and boundary conditions
Voltage clamp fluorometry	Invasive	Single-cell level	Research (electrophysiology)	Combines electrical and optical methods; used only in advanced research
Dynamic clamp	Invasive	Single-cell level	Research (electrophysiology)	Technically complex; not applicable to clinical settings
Two-electrode voltage clamp	Invasive	Single-cell level	Research (electrophysiology)	Useful for large cells, such as oocytes, but not for routine or clinical use

**Table 2:** Overview of disease-specific alterations in *C_m_* and associated clinical interpretations.

Author	Disease	*C_m_*
Małecka-Massalska et al. [26]	Head and neck cancer	*C_m_* was significantly higher in well-nourished patients compared to malnourished ones.
Barry et al. [27]	Healthy premenopausal women	*C_m_* showed considerable inter-individual variability but high intra-individual precision.
Yamada et al. [28]	Healthy individuals (skeletal muscle function)	*C_m_* positively correlated with muscle strength and contractile properties; higher *C_m_* was associated with greater torque production.
Cornish et al. [29]	Lymphedema	*C_m_* was higher in affected limbs compared to controls; values varied by limb and dominance.
Yashiro & Kotera, 2021 [30]	Haemodialysis	*C_m_* was lower after dialysis.
Sobieszek et al. [31]	Chronic heart failure with cachexia	*C_m_* was lower in advanced disease stages and negatively correlated with inflammatory markers.
Sobieszek et al. [32]	Chronic heart failure	*C_m_* was lower in advanced stages in men (significant) and in women (non-significant); negatively correlated with C-reactive protein in men.
Brantlov et al. [33]	Nephrotic syndrome	*C_m_* was lowest during active disease, higher in remission, and intermediate in healthy controls.
Popiołek et al. [34]	Anorexia nervosa	Higher *C_m_* was found in younger patients, with improvement during refeeding.
Cox-Reijven et al. [35]	Gastrointestinal disease	*C_m_* decreased with increasing severity of weight loss.

**Table 3:** Key bioimpedance parameters used for studying cellular properties with presentation of their respective calculations, definitions, roles in cell analysis, critical differences from *C_m_*, and suggestions for practical clinical applications.

Parameter	Formula	Definition	Function and Use	Features	Limitations
*C_m_* (nF)	$C_{m}=\frac{1}{2\pi f_{c}\left(R_{E}+R_{I}\right)}$	Cell membrane capacitance.	Reflects membrane health, structural integrity, function, and total membrane area; useful for detecting changes in membrane properties	Makes impedance measurement frequency-dependent	Measurement requires BIS device (cannot be measured with SF-BIA or MF-BIA)
*PhA* (degrees)	$PhA={tan}^{-1}\left(\frac{X_{C}}{R}\right)$	Phase shift between voltage and current.	Indicates cell mass, hydration, and membrane health; helpful for assessing overall tissue and fluid status	Most often reported at 50 kHz fixed frequency; it combines resistive and reactive impedance	Single-frequency (50 kHz) measurement gives limited information
*X_C_* (Ω)	$X_{C}=Im(Z_{TOTAL})$	Reactance is imaginary part of complex impedance *Z*	Indicates capacitive properties of cell membrane	Most often reported for fixed frequency of 50 kHz	Single-frequency (50 kHz) measurement gives limited information

*C_m_*: Cell membrane capacitance; *PhA*: phase angle; *X_C_*: capacitive reactance; AC: alternating current; *ω* = 2π*f*: angular frequency of applied AC signal.

## Data Availability

All data are presented in this paper.

## Abbreviations

The following abbreviations are used in this manuscript:

AC	Alternating current
BIA	Bioelectrical impedance analysis
BIS	Bioimpedance spectroscopy
*C_m_*	Cell membrane capacitance
CPE	Constant phase element
ICW	Intracellular water
MF-BIA	Multi-frequency bioelectrical impedance analysis
*PhA*	Phase angle
*R_E_*	Resistance of the extracellular water
*R_I_*	Resistance of the intracellular water
*R_INF_* (*R*_∞_)	Resistance at infinite frequency (*f* = ∞)
*R*_0_ (*R_E_*)	Resistance at zero frequency (*f* = 0)
SF-BIA	Single-frequency bioelectrical impedance analysis
TBW	Total body water
*X_C_*	Capacitive reactance
